# Domestic Economy in Hospitals

**Published:** 1894-11-10

**Authors:** 


### DOMESTIC ECONOMY IN HOSPITALS.

Mr. Thomas Ryan, Secretary to St. Mary's Hospital, W., writes: There is something seriously wrong in your "Institutional Workshop," hospital finance department, domestic economy section. The artisan there has been at work on St. Mary's Hospital accounts, head 1—provisions, with most astonishing results, and the work having been executed with the grossest inaccuracy, the workman proceeds with a highly *ex cathedra* air to dogmatically assert that the authorities of St. Mary's can hardly fail to be puzzled by the results of his handiwork, but are provided at once with a definite point for investigation. All this obviously greatly prejudices this hospital in the eyes of the public, and nothing but the strictest and most unimpeachable accuracy could justify its being published far and wide in such an influential paper as The Hospital.

This is what your "Institutional Workshop" artificer says (see your issue of October 13th): "St. Mary's provisions cost £32 a bed, as against £18 at University; and three times the amount of eggs and butter [cost, of course, is meant] is consumed per bed at St. Mary's than at University, and one-third more than at Middlesex." It will be sufficient for me to deal with these statements as affecting this hospital compared with Middlesex. The two institutions are more of a size than is the case with St. Mary's and University, and there are other reasons, unnecessary to state, why I select the comparison with Middlesex.

Now, sir, I wish to point out that your "Institutional Workshop" workman does not understand the use of his tools. He should be master of the "Hospital Annual," and be skilled in the correct use of a hospital report. The use of the "Hospital Annual" would have made him aware (see page cclvii.) that Middlesex supply their patients with no butter, sugar, or tea. These articles—for patients' use—cost St. Mary's £477; substract that sum from the total of provisions, £8,098, leaves £7,621. The extraordinary part of it is that the "Institutional Workshop" discussed this very point a fortnight before.

The use of a hospital report would have shown in the case of that of the Middlesex Hospital that on page 21 there stand these words, "In the Provision Account . . . . . is a substantial diminution of £516, mainly due to the transfer of officers to the Residential College" (a large percentage of this would be cheesemongery). Substract this also from St. Mary's figure and we get £7,621—£516—£7,105, as against £7,255 at Middlesex, no great difference be it observed, but what difference there is is in favour of St. Mary's.

On page 17 he would have found that three surgical wards had been entirely closed for three months. A study of the reports of both hospitals would also have informed him of the fact that while Middlesex is the larger hospital by thirty-nine beds, its in-patient work is less by over 800 patients; that therefore it works at less pressure, a fact which might well exercise a modifying effect on the expenditure. I am sure you will agree with me that accuracy is of supreme importance when the statements affect the reputation of a great medical charity.

*** We publish Mr. Ryan's argument as it stands. Anyone, who refers to the article in question, which appeared in The Hospital, pp. 31-32, for October 13th, will see that the figures are correctly given by the writer as taken from the audited accounts of the hospital in question. After careful reading and study Mr. Ryan throws a new light upon the meaning of these figures, and has so explained the difficulties they presented to the writer of the article in question. In future those who investigate hospital accounts must ascertain when a residential medical college is attached to a hospital whether the resident staff are accommodated there under a different system of accounts or not.—[Ed. *T. H.*]

